# Afferent and efferent projections of the rostral anterior cingulate cortex in young and middle-aged mice

**DOI:** 10.3389/fnagi.2022.960868

**Published:** 2022-08-17

**Authors:** Xinyi Ma, Wei Yu, Ping’an Yao, Yichen Zhu, Jiale Dai, Xiaofen He, Boyu Liu, Chi Xu, Xiaomei Shao, Jianqiao Fang, Zui Shen

**Affiliations:** Key Laboratory of Acupuncture and Neurology of Zhejiang Province, Department of Neurobiology and Acupuncture Research, The Third Clinical Medical College, Zhejiang Chinese Medical University, Hangzhou, China

**Keywords:** rostral ACC, afferent projections, efferent projections, young mice, middle-aged mice

## Abstract

Research shows that across life, the incidence of mental illness is highest in the young. In the context of the COVID-19 pandemic, mental health issues of the young in particular have received global attention. The rostral anterior cingulate cortex (rACC) plays an important role in psychiatric disorders and chronic pain-psychiatric comorbidities. However, it remains unknown whether or how the afferent and efferent circuits of the rACC change with aging. In this study, we microinjected a retrograde tracer virus and an anterograde trans-monosynaptic virus into the rACC of young and middle-aged mice (both male and female), and systematically and quantitatively analyzed the whole-brain afferent and efferent connections of rACC at different ages and sexes. Notably, in young and middle-aged mice, afferents of the rACC belong to four groups of brain structures arising mainly from the amygdala [mainly basolateral amygdaloid nucleus (BLA)] and cerebral cortex (mainly orbital cortex), with a small part originating from the basal forebrain and thalamus. In contrast, efferents of the rACC belong to four groups of brain structures mainly projecting to the thalamus (mainly ventral anterior-lateral/ventromedial thalamic nucleus (VAL/VM)], with a very small part projecting to the amygdala, basal forebrain, and cerebral cortex. Compared with young mice, the BLA-rACC circuit in middle-aged mice (male and female) did not change significantly, while the rACC-VAL/VM circuit in middle-aged mice (male and female) decreased significantly. In conclusion, this study comprehensively analyzed the input-output neural projections of rACC in mice of different ages and sexes and provided preliminary evidence for further targeted research.

## Introduction

Recently, a large research team led by Bethlehem and Seidlitz reported on standardized charts for the development of the human brain, which aided the identification of pathological changes in the brain (Bethlehem et al., 2022). This indicated that the largest case-control differences across epochs occurred in the young when the risk of mental disorders increased. In addition, since the outbreak of COVID-19, an approach that advocates home isolation to curb the spread of the virus has led to widespread mental health disorders globally; however, remarkably, the young were more affected by major depressive and anxiety disorders than the older ones (Santomauro et al., 2021). Therefore, more attention should be paid to mental disorders in the young.

As part of the prefrontal cortex, the rostral anterior cingulate cortex (rACC) is closely related to psychiatric disorders (including anxiety and depressive disorders) (Korb et al., 2009; Swartz et al., 2014; Suffren et al., 2019; Whitton et al., 2019) and mental illness induced by other diseases, such as chronic pain (Gomtsian et al., 2018; Shen et al., 2020). Although there is evidence indicating rACC neural structural modifications with aging (Stranahan et al., 2012), it remains unknown whether or how the afferent and efferent circuits of the rACC change with age.

In previous studies, to observe the efferents of a certain brain area, non-transsynaptic anterograde tracer viruses were often used; however, this method can only label the axons or dendrites (fibers) of neurons, but cannot determine whether there is transmission to the postsynaptic neurons in the brain region with fiber distribution (maybe the fibers just pass through a certain brain area). To avoid the above situation, in this study, we adapted an anterograde trans-monosynaptic virus to observe the efferent circuits of rACC by quantitatively analyzing the number of trans-monosynaptic-labeled neuronal somata.

In this study, we microinjected a retrograde tracer virus and an anterograde trans-monosynaptic virus into the rACC brain regions of young and middle-aged mice (both male and female), and systematically and quantitatively analyzed whole-brain afferent and efferent connections of the rACC at different ages and sexes.

## Materials and methods

### Animals

C57BL/6J mice were used in this study, with four mice in each group of 7-week-old male young mice weighing 22–25 g, 7-week-old female young mice weighing 20–25 g, 11-month-old male middle-aged mice weighing 31–33 g, and 11-month-old female middle-aged mice weighing 26–32 g. The animals were kept in a standard feeding environment at room temperature (24 ± 1°C) and humidity (50 ± 10%), with free access to food and water, a 12 h light cycle, and proper ventilation. Each group of mice was housed in cages, and the bottom of each cage was covered with sterile pine padding to protect the toes. All animal disposals during the experiment were in accordance with the Guidance Suggestion of Caring for Laboratory Animals, promulgated by the Ministry of Science and Technology of the People’s Republic of China in 2006.

### Virus and trace injection

A retrograde non-transsynaptic adeno-associated virus (AAV) (AAV2/2-Retro-hSyn-EGFP, PT-1990, BrainVTA, China) with a viral titer of 1.05 × 10^13^ vg/mL was used as a retrograde tracer in this study, while an anterograde monosynaptic AAV (AAV2/1-hSyn-CRE-mCherry, PT-0407, BrainVTA, China) with a viral titer of 1.20 × 10^13^ vg/mL was used as an antegrade tracer.

Experimental mice were anesthetized by intraperitoneal injection of 0.3% sodium pentobarbital. The anesthetized mice were skinned, sterilized, and fixed on a stereotaxic apparatus (68025, RWD, China) with a mouse adapter (68030, RWD, China). Heating pads (Temperature controller 69000, RWD, China) were used to control the temperature to maintain the body temperature of the mice during the procedure. The rACC (AP, + 1.50 mm; ML, ± 0.35 mm; DV, −0.85 mm) was injected with 80 nL viruses at a speed of 40 nL⋅min^–1^ using a micropump (Legato 130, KD Scientific, United States) assembled with glass microelectrodes. The micropump was left in place for 10 min after the injection to avoid virus spillage from the injection site. The incision was then sutured and sterilized. After the operation, the animals were placed on heating pads and returned to the cage after they regained consciousness and normal activities.

### Tissue preparation, immunohistochemistry, and imaging of brain slices

Five weeks after virus injection, the mice were anesthetized with 0.3% sodium pentobarbital and then intravenously infused with 4°C normal saline and 4% paraformaldehyde sequentially, through the heart. The brain tissue was removed, post-fixed with 4% paraformaldehyde, and then dehydrated in sequence along a concentration gradient with 15 and 30% sucrose solutions.

Coronal brain slices (30 μm) were cut using a cryostat (CryoStar NX50, Thermo Fisher Scientific, United States) and mounted with a fluorescent medium containing DAPI (ab104139, Abcam, United States). All brain slices were imaged using a virtual slide microscope (VS120-S6-W, Olympus, Japan).

### Data analysis and iconography

Compared with the brain atlas of mice (Paxinos and Franklin, 2019), we checked the location of virus injection, and the location of rACC virus injection for each mouse is represented in the study. The fluorescence intensity at the injection site was determined using ImageJ software to represent the expression level of the virus.

Five slices were evenly selected from the projection site of the brain, and the number of positive cells at the projection site on the selected slices was counted using the ImageJ software. The value obtained by multiplying the average value of the five brain slices by the total number of brain slices in this brain area was taken as the total number of positive cells in this projection brain area.

When comparing different ages and sexes, the total number of positive cells at the projection site was divided by the normalized fluorescence intensity at the injection site, to avoid the influence of different injected virus expression levels on projection-positive cells.

Data are expressed as mean ± s.e.m. and were analyzed using OriginPro software (version 9.4, OriginLab Corporation, United States). Data from Figures 5–7 and Supplementary Figures 1, 2 were analyzed using two-way ANOVA (with age and sex as factors) with Fisher LSD *post-hoc* analysis.

## Results

In the third edition and previous mouse brain atlas (Paxinos and Franklin, 2001, 2007), the rACC is located on the rostral Cg1 (anterior to the junction of the left and right corpus callosum). In the fourth and fifth editions of the mouse brain atlas (Paxinos and Franklin, 2013, 2019), the rACC is located on the rostral A24b region (anterior to the junction of the left and right corpus callosum). In this study, we used the fifth edition of the mouse brain atlas (Paxinos and Franklin, 2019), to determine the location of brain regions including rACC.

### Retrograde and anterograde adeno-associated virus injection site

**FIGURE 1:**
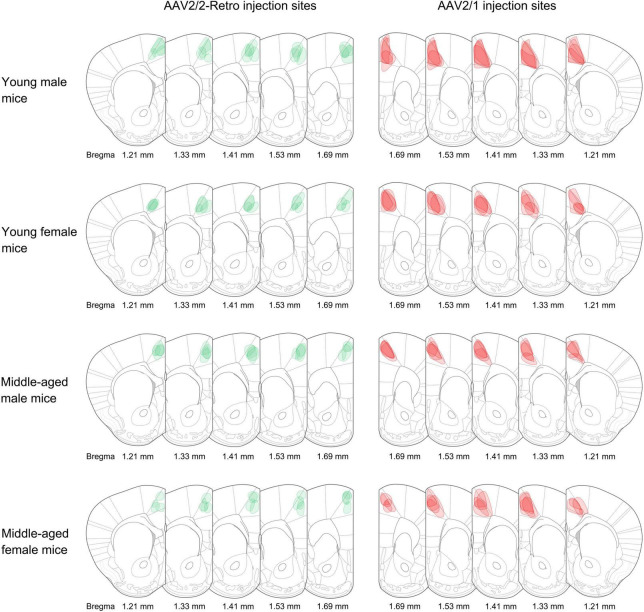
Adeno-associated virus (AAV) injection sites. The schematic depicts the actual injection site of the virus into the rACC. The boundaries of the green and red colors represent the range of retrograde non-transsynaptic (AAV2/2-Retro-hSyn-EGFP) and anterograde monosynaptic (AAV2/1-hSyn-CRE-mCherry) AAV. The corresponding bregma value of brain slices is marked below. The image represents *n* = 4 animals.

Retrograde non-transsynaptic (green fluorescent marker) and anterograde monosynaptic (red fluorescent marker) AAV were injected into the rACC. Four young males, four middle-aged males, four young females, and four middle-aged females were selected for data analysis. The boundary with the same color as the virus was used to describe the range of expression at the corresponding bregma level (Figure 1).

Table 1 classifies these brain regions.

**TABLE 1:** Brain region classification.

Brain region	Subarea	Structures	Bregma range
Amygdala	BLA	BLAa	−0.59 mm to −2.03 mm
Basal forebrain	CLA	CLA, DCL, VCL	+1.69 mm to +1.07 mm
Cerebral cortex	AIC	AID, AIV	+2.33 mm to + 0.49 mm
	OC	MO, VO, LO, DLO	+3.17 mm to +1.69 mm
Thalamic anterior group	AM		−0.47 mm to −1.23 mm
	IAM		−0.71 mm to −1.31 mm
Thalamicmedial group	MD	MD, MDC, MDL, MDM	−0.59 mm to −2.03 mm
Thalamic lateral group	LD	LDVL, LDDM	−0.83 mm to −1.67 mm
	LPM	LPMC, LPMR	−1.43 mm to −3.15 mm
Thalamic ventral group	VAL,VM	VAL, VM	−0.59 mm to −2.15 mm
	VPL,VPM	VPL, VPM	−0.83 mm to −2.53 mm
Thalamic intralaminar group	CL		−1.07 mm to −2.15 mm
	CM		−0.47 mm to −2.03 mm
	PC		−0.47 mm to −2.03 mm
Thalamic midline group	PVT	PV, PVA, PVP	−0.23 mm to −2.27 mm
Thalamic posterior group	Ang		−1.23 mm to −1.55 mm
	Po		−1.31 mm to −2.69 mm

### Afferents and efferents of the rostral anterior cingulate cortex in mice of the same age and sex

We observed the distribution of rACC input-output neuronal circuits in different brain regions of young male mice, middle-aged male mice, young female mice, and middle-aged female mice. We divided them into six categories according to the density of the projection cells: heavy labeling (*n* ≥ 500), dense labeling (100 ≤ n < 500), moderate labeling (50 ≤ n < 100), light labeling (10 ≤ n < 50), sporadic labeling (0 < n < 10), and no labeling.

**FIGURE 2:**
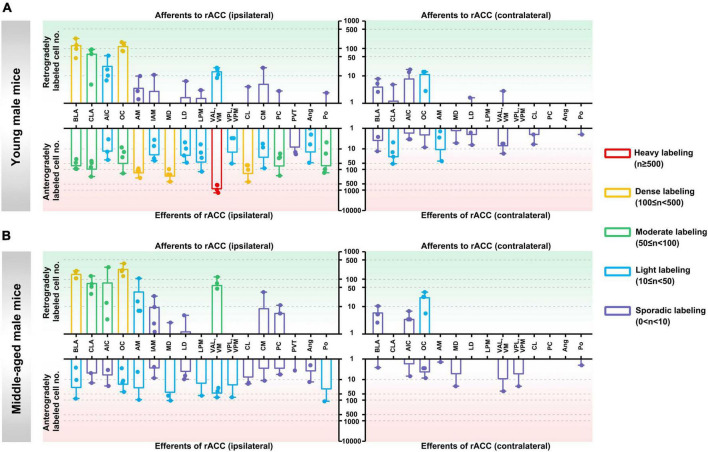
Afferents and efferents of the rACC in young and middle-aged male mice. **(A)** The number of retrogradely and anterogradely labeled cells in different brain regions ipsilateral and contralateral to the injection site of the rACC was quantified in young male mice (*n* = 3–4 animals, error bars represent s.e.m.). **(B)** The number of retrogradely and anterogradely labeled cells in different brain regions ipsilateral and contralateral to the injection site of rACC was quantified in middle-aged male mice (*n* = 3–4 animals, error bars represent s.e.m.).

**In the ipsilateral input brain regions of the rACC in young male mice,** the basolateral amygdaloid nucleus (BLA) and orbital cortex (OC) were densely labeled, the claustrum (CLA) was moderately labeled, and the agranular insular cortex (AIC) and ventral anterior-lateral/ventromedial thalamic nucleus (VAL/VM) were lightly labeled. The anteromedial thalamic nucleus (AM), interanteromedial thalamic nucleus (IAM), laterodorsal thalamic nucleus (LD), lateral posterior thalamic nucleus—medial part (LPM), centrolateral thalamic nucleus (CL), central medial thalamic nucleus (CM), paracentral thalamic nucleus (PC), and posterior thalamic nuclear group (Po) were sporadically labeled, while the ventral posterolateral/ventral posteromedial thalamic nucleus (VPL/VPM), paraventricular thalamic nucleus (PVT), and angular thalamic nucleus (Ang) were not labeled (Figure 2A, upper left part).

**In the contralateral input brain regions of the rACC in young male mice,** the OC was lightly labeled, whereas the BLA, CLA, AIC, LD, and VAL/VM were sporadically labeled. The remaining brain regions were not labeled (Figure 2A, upper right part).

**In the ipsilateral output brain regions of the rACC in young male mice,** the VAL/VM was heavily labeled; the AM, mediodorsal thalamic nucleus (MD), and CL were densely labeled; the BLA, CLA, OC, PC, and Po were moderately labeled; the AIC, IAM, LD, LPM, VPL/VPM, CM, and Ang were light labeled; and the PVT was sporadically labeled (Figure 2A, lower left part).

**In the contralateral output brain regions of the rACC in young male mice,** the CLA and AM were lightly labeled; the BLA, AIC, OC, MD, LD, VAL/VM, CL, and Po were sporadically labeled; and the LPM, VPL/VPM, PC, and Ang were not labeled (Figure 2A, lower right part).

**In the ipsilateral input brain regions of the rACC in middle-aged male mice,** the BLA and OC were densely labeled; the CLA, AIC, and VAL/VM were moderately labeled; the AM was lightly labeled; the IAM, MD, LD, CM, and PC were sporadically labeled; and the LPM, VPL/VPM, CL, PVT, Ang, and Po were not labeled (Figure 2B, upper left part).

**In the contralateral input brain regions of the rACC in middle-aged male mice,** the OC was lightly labeled, and the BLA and AIC were sporadically labeled. The remaining brain regions were not labeled (Figure 2B, upper right part).

**In the ipsilateral output brain regions of the rACC in middle-aged male mice,** the BLA, OC, AM, MD, LPM, VAL/VM, VPL/VPM, and Po were lightly labeled; and the CLA, AIC, IAM, LD, CL, CM, PC, PVT, and Ang were sporadically labeled (Figure 2B, lower left part).

**In the contralateral output brain regions of the rACC in middle-aged male mice,** the BLA, AIC, OC, AM, MD, VAL/VM, VPL/VPM, and Po were sporadically labeled, while the remaining brain regions were not labeled (Figure 2B, lower right part).

**FIGURE 3:**
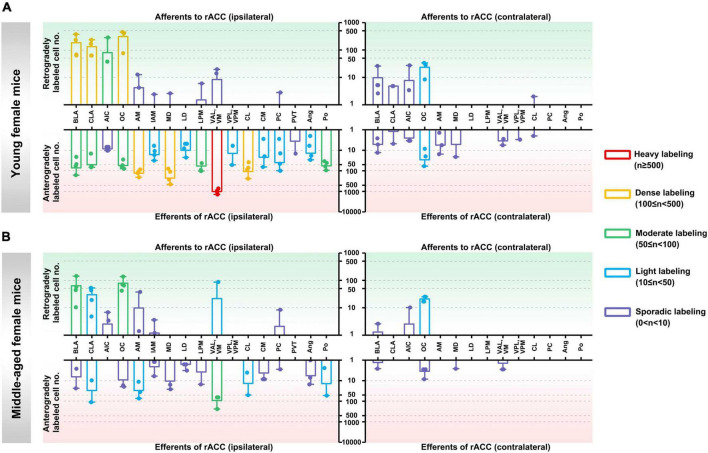
Afferents and efferents of the rACC in young and middle-aged female mice. **(A)** The number of retrogradely and anterogradely labeled cells in different brain regions ipsilateral and contralateral to the injection site of the rACC was quantified in young female mice (*n* = 3–4 animals, error bars represent s.e.m.). **(B)** The number of retrogradely and anterogradely labeled cells in different brain regions ipsilateral and contralateral to the injection site of the rACC was quantified in middle-aged female mice (*n* = 3–4 animals, error bars represent s.e.m.).

**In the ipsilateral input brain regions of the rACC in young female mice,** the BLA, CLA, and OC were densely labeled; the AIC was moderately labeled; the AM, IAM, MD, LPM, VAL/VM, and PC were sporadically labeled; and the LD, VPL/VPM, CL, CM, PVT, Ang, and Po were not labeled (Figure 3A, upper left part).

**In the contralateral input brain regions of the rACC in young female mice,** the OC was lightly labeled, and the BLA, CLA, AIC, and CL were sporadically labeled. The remaining brain regions were not labeled (Figure 3A, upper right part).

**In the ipsilateral output brain regions of the rACC in young female mice,** the VAL/VM was heavily labeled; the AM, MD, and CL were densely labeled; the BLA, CLA, OC, LPM, and Po were moderately labeled; the IAM, LD, VPL/VPM, CM, PC, and Ang were lightly labeled; and the AIC and PVT were sporadically labeled (Figure 3A, lower left part).

**In the contralateral output brain regions of the rACC in young female mice,** the OC was lightly labeled; the BLA, CLA, AIC, AM, MD, VAL/VM, VPL/VPM, and CL were sporadically labeled; and the LD, LPM, PC, Ang, and Po were not labeled (Figure 3A, lower right part).

**In the ipsilateral input brain regions of the rACC in middle-aged female mice,** the BLA and OC were moderately labeled; the CLA and VAL/VM were lightly labeled; the AIC, AM, IAM, and PC were sporadically labeled; and the MD, LD, LPM, VPL/VPM, CL, CM, PVT, Ang, and Po were not labeled (Figure 3B, upper left part).

**In the contralateral input brain regions of the rACC in middle-aged female mice,** the OC was lightly labeled, and the BLA and AIC were sporadically labeled. The remaining brain regions were not labeled (Figure 3B, upper right part).

**In the ipsilateral output brain regions of the rACC in middle-aged female mice,** the VAL/VM was moderately labeled; the CLA, AM, CL, and Po were lightly labeled; the BLA, OC, IAM, MD, LD, LPM, CM, PC, and Ang were sporadically labeled; and the AIC, VPL/VPM, and PVT were not labeled (Figure 3B, lower left part).

**In the contralateral output brain regions of the rACC in middle-aged female mice,** the BLA, OC, MD, and VAL/VM were sporadically labeled, while the remaining brain regions were not labeled (Figure 3B, lower right part).

Overall, the input-output neuronal circuits of the rACC are mainly distributed on the ipsilateral side, with a small amount distributed on the contralateral side. The input brain regions of the rACC are mainly concentrated in the amygdala, basal forebrain, and cerebral cortex, whereas their distribution in each region of the thalamus is relatively sparse. The output brain regions of the rACC are mainly distributed in various regions of the thalamus.

### Weight distribution of retrogradely and anterogradely labeled cells of the rostral anterior cingulate cortex in different brain regions

We further adopted a tree map to observe the weight distribution of retrogradely and anterogradely labeled cells of the rACC in different brain regions.

**FIGURE 4:**
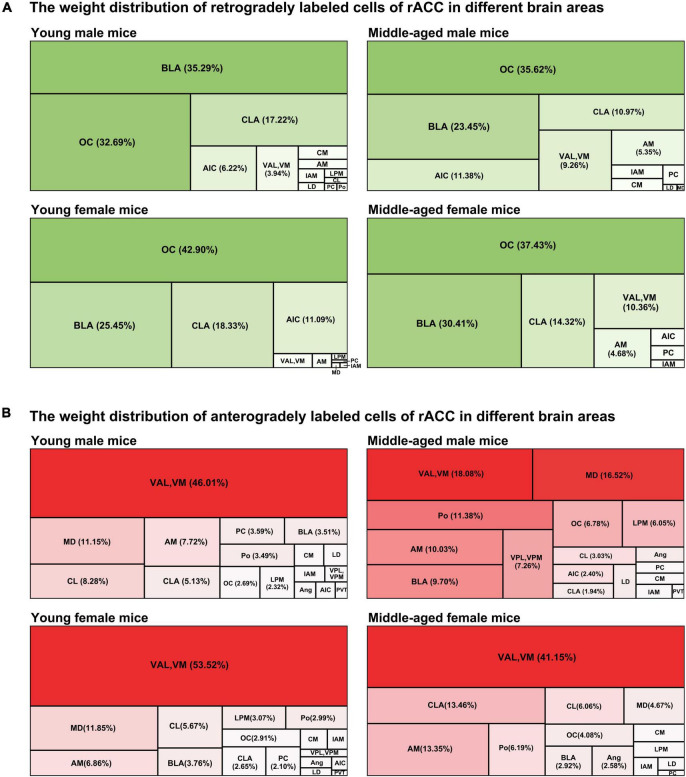
The weight distribution of retrogradely and anterogradely labeled cells of the rACC in different brain regions. **(A)** The four tree maps indicate the weight distribution of retrogradely labeled cells of the rACC in different brain areas. **(B)** The four tree maps indicate the weight distribution of anterogradely labeled cells of the rACC in different brain areas.

In the input brain regions of the rACC in mice of different ages and sexes, the BLA and OC were the major components, while the CLA was a minor component. The AIC was a minor component in young female mice, while the VAL and VM were minor components in middle-aged female mice (Figure 4A).

**FIGURE 5:**
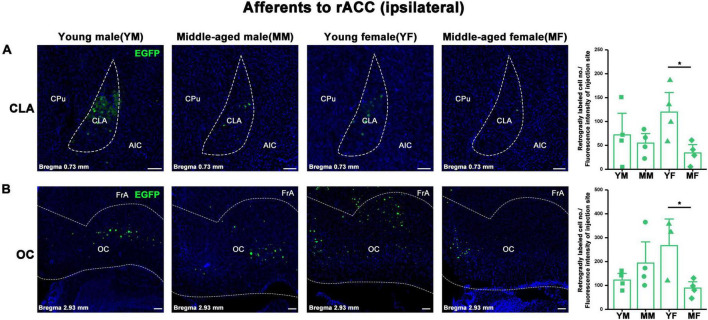
Ipsilateral brain regions with retrograde projections of the rACC in mice of different ages and sexes **(A,B)** Left: Representative images of retrogradely labeled neurons in the CLA; OC regions of young male, middle-aged male, young female, and middle-aged female mice. Right: Retrogradely labeled cell number divided by fluorescence intensity of injection site (*n* = 4 young males, *n* = 4 middle-aged males, *n* = 4 young females in the CLA, *n* = 3 young females in the OC,*n* = 4 middle-aged females). *p < 0.05. Scale bar = 100 μm. Error bars represent s.e.m.

**FIGURE 6:**
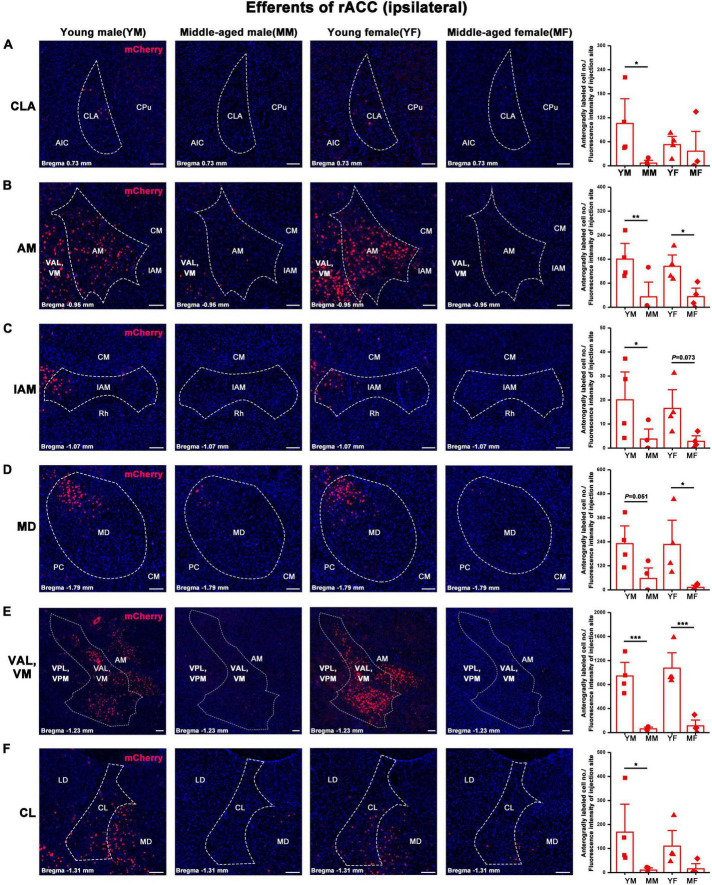
Ipsilateral brain regions with anterograde projections of the rACC in mice of different ages and sexes. **(A–F)** Left: Representative images of anterogradely labeled neurons in the CLA, AM, IAM, MD, VAL, VM, and CL regions of young male, middle-aged male, young female, and middle-aged female mice. Right: Anterogradely labeled cell number divided by fluorescence intensity of injection site (*n* = 4 young males, *n* = 4 middle-aged males, *n* = 4 young females, *n* = 4 middle-aged females).*p < 0.05, **p < 0.01, ***p < 0.001. Scale bar = 100 μm. Error bars represent s.e.m.

**FIGURE 7:**
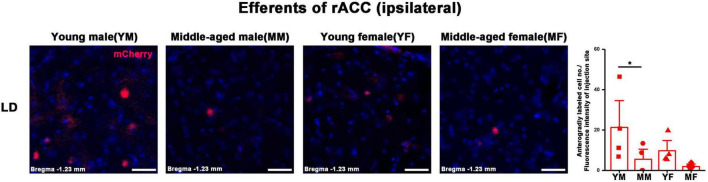
Ipsilateral brain regions with anterograde projections of the rACC in mice of different ages and sexes. Left: Representative images of anterogradely labeled neurons in the LD regions of young male, middle-aged male, young female, and middle-aged female mice. Right: Anterogradely labeled cell number divided by fluorescence intensity of injection site (*n* = 4 young males, *n* = 4 middle-aged males, *n* = 4 young females, *n* = 4 middle-aged females). *p < 0.05. Scale bar = 20 μm. Error bars represent s.e.m.

The VAL/VM was the major component of the output brain regions of the rACC in young male mice, young female mice, and middle-aged female mice (all > 40%). The VAL and VM, which accounted for only approximately 18.08% of all output projections of the rACC in middle-aged male mice, became minor output brain areas. The MD is a minor part of the output brain regions of the rACC in young male mice, middle-aged male mice, and young female mice. The Po, AM, and BLA are minor parts of the output brain area of the rACC in middle-aged male mice. The CLA and AM are minor parts of the output brain area of the rACC in middle-aged female mice (Figure 4B).

### Comparison of different ages and sexes in the same afferents and efferents of the rostral anterior cingulate cortex

We further explored the brain regions that exhibited statistically different neuronal expressions in mice of different ages and sexes in the same afferents and efferents of the rACC. Although we used uniform parameters for virus injection and administered the same injection volume to each mouse, projection differences owing to virus infection efficiency could not be avoided. Therefore, we determined the fluorescence intensity at the injection site, and the number of labeled neurons in the input or output brain areas was divided by the fluorescence intensity at the corresponding AAV injection site. This normalized value was used for statistical comparison.

In the ipsilateral input brain areas of the rACC, the number of labeled neurons in the CLA of middle-aged female mice was significantly lower than that of young female mice (two-way ANOVA: F_age[1,12]_ = 5.365, *p* = 0.039; *post-hoc* Fisher LSD: middle-aged female versus young female, t_12_ = −2.731, *p* = 0.018, Figure 5A). Similarly, the number of labeled neurons in the OC of middle-aged female mice was significantly lower than that of young female mice (two-way ANOVA: F_age[1,11]_ = 1.396, *p* = 0.262, F_interaction[1,11]_ = 7.631, *p* = 0.018; *post-hoc* Fisher LSD: middle-aged female versus young female, t_11_ = −2.687, *p* = 0.021, Figure 5B). No statistical difference was found in the rest of the ipsilateral input brain regions of the rACC (Supplementary Figure 1A). The detailed statistical comparison of the contralateral input brain regions of the rACC is shown in Supplementary Figure 2A.

The number of positive cells in the output brain regions of the rACC was statistically different for CLA, AM, IAM, MD, VAL/VM, CL, and LD (Figures 6, 7).

In the ipsilateral output brain area of the rACC, the number of labeled neurons in the CLA of middle-aged male mice was significantly lower than that of young male mice (two-way ANOVA: F_age[1,12]_ = 4.454, *p* = 0.056; *post-hoc* Fisher LSD: middle-aged male versus young male, t_12_ = −2.562, *p* = 0.025, Figure 6A). The number of labeled neurons in the AM of middle-aged male and female mice was significantly lower than that of young mice of the same sex (two-way ANOVA: F_age[1,12]_ = 15.804, *p* = 0.002; *post-hoc* Fisher LSD: middle-aged male versus young male, t_12_ = −3.120, *p* = 0.009; middle-aged female versus young female, t_12_ = −2.502, *p* = 0.028, Figure 6B). The number of labeled neurons in the IAM of middle-aged male mice was significantly lower than that of young male mice (two-way ANOVA: F_age[1,12]_ = 9.322, *p* = 0.010; *post-hoc* Fisher LSD: middle-aged male versus young male, t_12_ = −2.350, *p* = 0.037; middle-aged female versus young female, t_12_ = −1.968, *p* = 0.073, Figure 6C). The number of labeled neurons in the MD of middle-aged male and female mice was significantly lower than that in young mice of the same sex (two-way ANOVA: F_age[1,12]_ = 12.290, *p* = 0.004; *post-hoc* Fisher LSD: middle-aged male versus young male, t_12_ = −2.172, *p* = 0.051; middle-aged female versus young female, t_12_ = −2.786, *p* = 0.016, Figure 6D). The number of labeled neurons in the VAL/VM of middle-aged male and female mice was significantly lower than that of young mice of the same sex (two-way ANOVA: F_age[1,11]_ = 51.685, *p* < 0.001; *post-hoc* Fisher LSD: middle-aged male versus young male, t_11_ = −4.677, *p* < 0.001; middle-aged female versus young female, t_11_ = −5.519, *p* < 0.001, Figure 6E). The number of labeled neurons in the CL of middle-aged male mice was significantly lower than that of young male mice (two-way ANOVA: F_age[1,12]_ = 7.825, *p* = 0.016; *post-hoc* Fisher LSD: middle-aged male versus young male, t_12_ = −2.480, *p* = 0.029, Figure 6F). The number of labeled neurons in the LD of middle-aged male mice was significantly lower than that of young male mice (two-way ANOVA: F_age[1,12]_ = 5.464, *p* = 0.038; *post-hoc* Fisher LSD: middle-aged male versus young male, t_12_ = −2.208, *p* = 0.047, Figure 7).

No statistical difference was found in the rest of the ipsilateral output brain regions of the rACC (Supplementary Figure 1B), and a detailed statistical comparison of the contralateral output brain regions of the rACC is shown in Supplementary Figure 2B.

## Discussion

**FIGURE 8:**
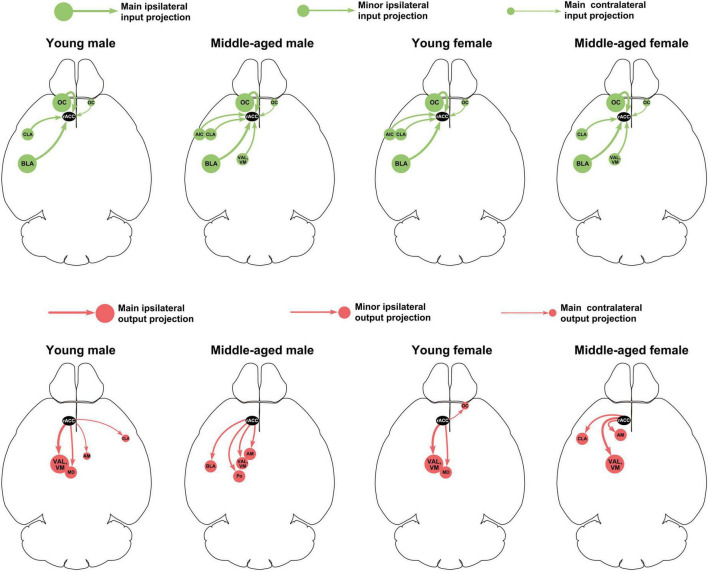
Summary of main and minor input-output projection circuits of the rACC in the bilateral brain of mice of different ages and sexes.

This study provides a detailed analysis of the whole-brain input-output neuronal circuit of the rACC in mice of different ages (young and middle-aged) and sexes (male and female). Figure 8 summarizes the performance of the main and minor input-output projection circuits of the rACC in the bilateral brain of different ages and sexes. In young and middle-aged mice, the afferents of the rACC belong to four groups of brain structures arising mainly from the amygdala and cerebral cortex, with a small part originating from the basal forebrain and thalamus. In contrast, the efferents of the rACC belong to four groups of brain structures that mainly project to the thalamus, with a very small portion projecting to the amygdala, basal forebrain, and cerebral cortex.

In this study, we found that the BLA is the main input brain region of the rACC. The existence of a mutual projection between the rACC and BLA has also been definitively reported in literature (Kitamura et al., 2017). It should be noted that in this study, we only observed obvious virus-labeled positive projecting cells in the basolateral amygdaloid nucleus—anterior part (BLAa) (Supplementary Figure 3), and no obvious virus-labeled positive projecting cells were observed in other subregions of the amygdala [including the basolateral amygdaloid nucleus—posterior part (BLAp), lateral amygdaloid nucleus (LA), and central amygdaloid nucleus (CEA)]. The BLA is well documented to be involved in negative emotional processing, such as aversion (Molero-Chamizo, 2017; Jean-Richard-Dit-Bressel et al., 2022), anxiety (Duan et al., 2021; Zheng et al., 2021), and fear (Sah, 2017; Sun et al., 2020). In contrast, an increasing number of studies has indicated that the BLA is a key element in pain processing and comorbid pain and psychiatric disorders (Deyama et al., 2007; Li et al., 2013; Thompson and Neugebauer, 2017; Neugebauer et al., 2020).

The OC, another main input brain region of the rACC in this study, is mainly involved in the regulation of pain, psychiatric disorders, and pain-psychiatric comorbidities (Samara et al., 2018; Chu et al., 2020; Sheng et al., 2020). Our results show that the BLA-rACC and OC-rACC circuits do not change significantly with age (especially as regards the proportion of total projections). These indicate that, although the two circuits are closely related to emotional processing, they may not be the main circuits involved in the higher incidence of psychiatric disorders in the young compared with the middle-aged or elderly.

Our results show that the VAL/VM was the most dominant output-projection brain region of the rACC. Previous studies have shown that the VAL/VM belongs to the motor nucleus of the thalamus and is an internal brain area of the thalamus that encodes and integrates motor information (Gaidica et al., 2018; Di Giovanni et al., 2020). The VAL/VM is not only involved in the execution of regulated movements but also in their planning (Guo et al., 2017; Svoboda and Li, 2018). Negative emotions often lead to negative motor planning or selection, which in turn leads to negative behaviors (e.g., avoidance and reduced activity) (Corr, 2013; Donner and Lowry, 2013; Bystritsky et al., 2021). Our previous study showed that specific activation of the rACC-VAL circuit can produce anxiety-like behaviors, and specific inhibition of the rACC-VAL circuit can alleviate chronic pain-induced anxiety-like behaviors but does not affect pain sensitization (Shen et al., 2020). In the present study, in terms of the number of infected cells across monosynapses, the rACC-VAL/VM circuit significantly reduced in both male and female mice than in young and middle-aged mice. As regards the proportion of rACC efferents, the rACC-VAL/VM circuit of male middle-aged mice significantly decreased compared with that of young male mice, while the rACC-VAL/VM circuit in female middle-aged mice decreased less than in young mice. Therefore, we speculate that the higher incidence of negative emotions in young people than in middle-aged people may be closely related to the rACC-VAL/VM circuit, although more evidence is required in the future. The effect of menopause on negative emotions in middle-aged women compared to middle-aged men may be a reason why the rACC-VAL/VM circuit in middle-aged female mice did not significantly reduce.

Furthermore, in our study, the rACC-MD circuit decreased with age. In previous studies, the MD was found to be closely related to cognitive functions, such as memory and decision-making (Watanabe and Funahashi, 2012; Mitchell, 2015; Pysick et al., 2021), which suggests that the decline in cognitive functions, such as memory, may begin in middle age and may be related to the rACC-MD circuit.

In conclusion, this study comprehensively analyzed the input-output neural projections of the rACC in mice of different ages and sexes, and provided preliminary evidence for further targeted research.

## Data availability statement

The datasets presented in this article are not readily available because the raw data supporting the conclusions of this article will be made available by the authors, without undue reservation. Requests to access the datasets should be directed to ZS, shenzui1228@163.com.

## Ethics statement

The animal study was reviewed and approved by the Animal Ethics Committee of Zhejiang Chinese Medical University.

## Author contributions

XM performed the surgeries, data statistics, and manuscript writing. WY performed the immunostaining. PY contributed to the animal rearing. YZ and JD contributed to the tissue preparation. XH, BL, and CX contributed to the data interpretation. JF and XS contributed to the theoretical guidance. ZS performed the data analysis and wrote the manuscript. All authors contributed to the article and approved the submitted version.

## Supplementary material

Supplementary Figure 1 Ipsilateral brain regions with afferent and efferent projections of the rACC in mice of different ages and sexes. *n* = 4 young males, *n* = 4 middle-aged males, *n* = 4 young females, *n* = 4 young females, and *n* = 4 middle-aged females. Error bars represent s.e.m.

Supplementary Figure 2 Contralateral brain regions with afferent and efferent projections of the rACC in mice of different ages and sexes. *n* = 4 young males, *n* = 4 middle-aged males, *n* = 4 young females, *n* = 4 young females, and *n* = 4 middle-aged females. **p* < 0.05, ***p* < 0.01, ****p* < 0.001. Error bars represent s.e.m.

Supplementary Figure 3 Ipsilateral BLAa regions with afferent and efferent projections of the rACC in mice of different ages and sexes. Representative images of retrogradely and anterogradely labeled neurons in the BLAa from the rACC. Scale bar = 100 μm.

## Abbreviations

AIC: agranular insular cortex
AID: agranular insular cortex, dorsal part
AIV: agranular insular cortex, ventral part
AM: anteromedial thalamic nucleus
Ang: angular thalamic nucleus
BLA: basolateral amygdaloid nucleus
BLAa: basolateral amygdaloid nucleus, anterior part
BLAp: basolateral amygdaloid nucleus, posterior part
BLAv: basolateral amygdaloid nucleus, ventral part
CeA: central amygdaloid nucleus
CL: centrolateral thalamic nucleus
CLA: claustrum
CM: central medial thalamic nucleus
CPu: caudate putamen (striatum)
DCL: dorsal claustrum
DLO: dorsolateral orbital cortex
FrA: frontal association cortex
IAM: interanteromedial thalamic nucleus
La: lateral amygdaloid nucleus
LD: laterodorsal thalamic nucleus
LDDM: laterodorsal thalamic nucleus, dorsomedial part
LDVL: laterodorsal thalamic nucleus, ventrolateral part
LO: lateral orbital cortex
LPM: lateral posterior thalamic nucleus, medial part
LPMC: lateral posterior thalamic nucleus, mediocaudal part
LPMR: lateral posterior thalamic nucleus, mediorostral part
MD: mediodorsal thalamic nucleus
MDC: mediodorsal thalamic nucleus, central part
MDL: mediodorsal thalamic nucleus, lateral part
MDM: mediodorsal thalamic nucleus, medial part
MO: medial orbital cortex
OC: orbital cortex
PC: paracentral thalamic nucleus
Pir: piriform cortex
Po: posterior thalamic nuclear group
PVA: paraventricular thalamic nucleus, anterior part
PVP: paraventricular thalamic nucleus, posterior part
PVT: paraventricular thalamic nucleus
Rh: rhomboid thalamic nucleus
VAL: ventral anterior-lateral thalamic nucleus
VCL: ventral claustrum
VM: ventromedial thalamic nucleus
VO: ventral orbital cortex
VPL: ventral posterolateral thalamic nucleus
VPM: ventral posteromedial thalamic nucleus.
