# Supplementary figures and images for: Afferent and efferent projections of the rostral anterior cingulate cortex in young and middle-aged mice
Source: Front Aging Neurosci. 2022 Aug 17;14:960868. doi: 10.3389/fnagi.2022.960868 (PMC9428471; doi:10.3389/fnagi.2022.960868)

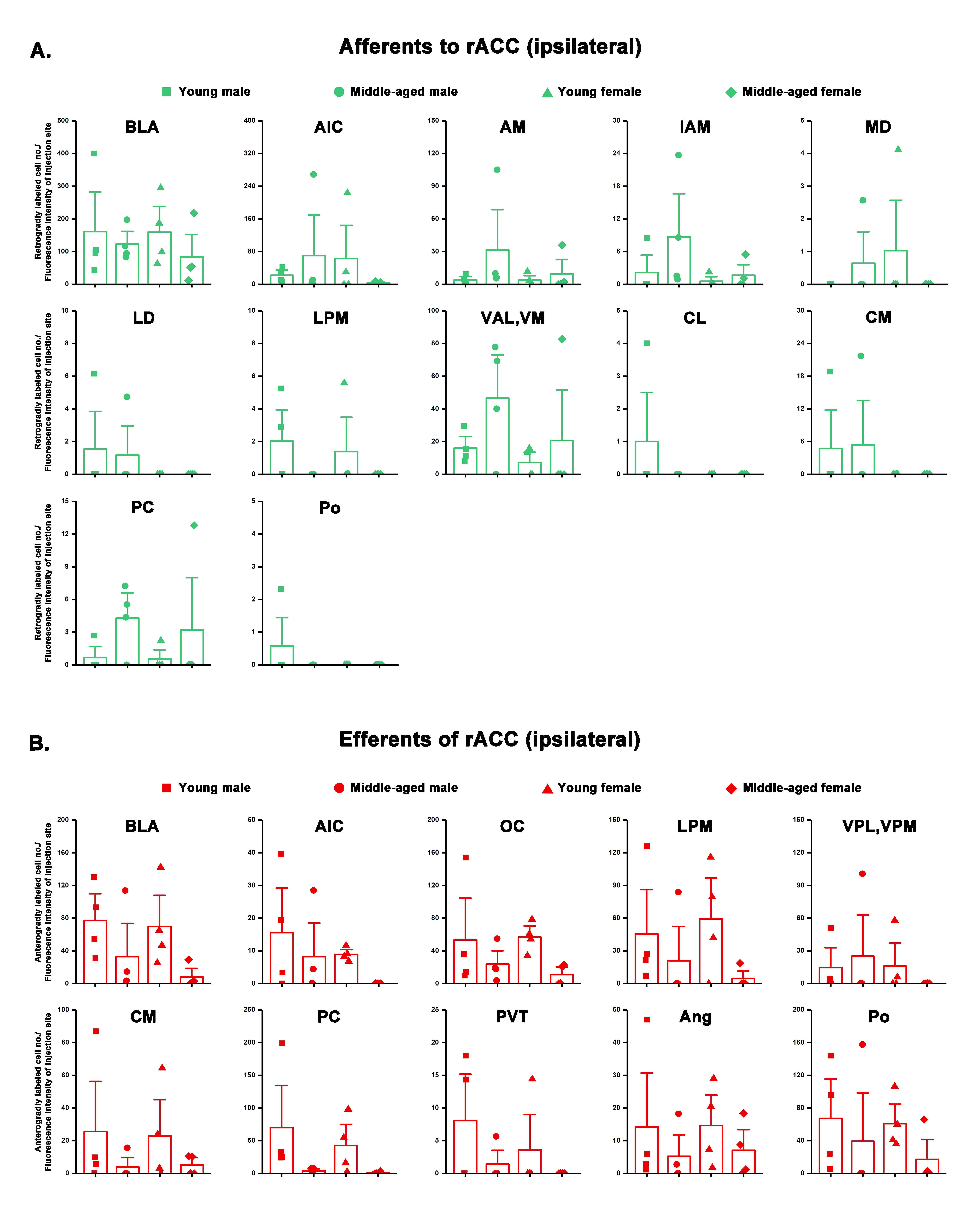

Supplement: Supplementary Figure 1 — Ipsilateral brain regions with afferent and efferent projections of the rACC in mice of different ages and sexes. n = 4 young males, n = 4 middle-aged males, n = 4 young females, n = 4 young females, and n = 4 middle-aged females. Error bars represent s.e.m. [file Image_1.jpg]

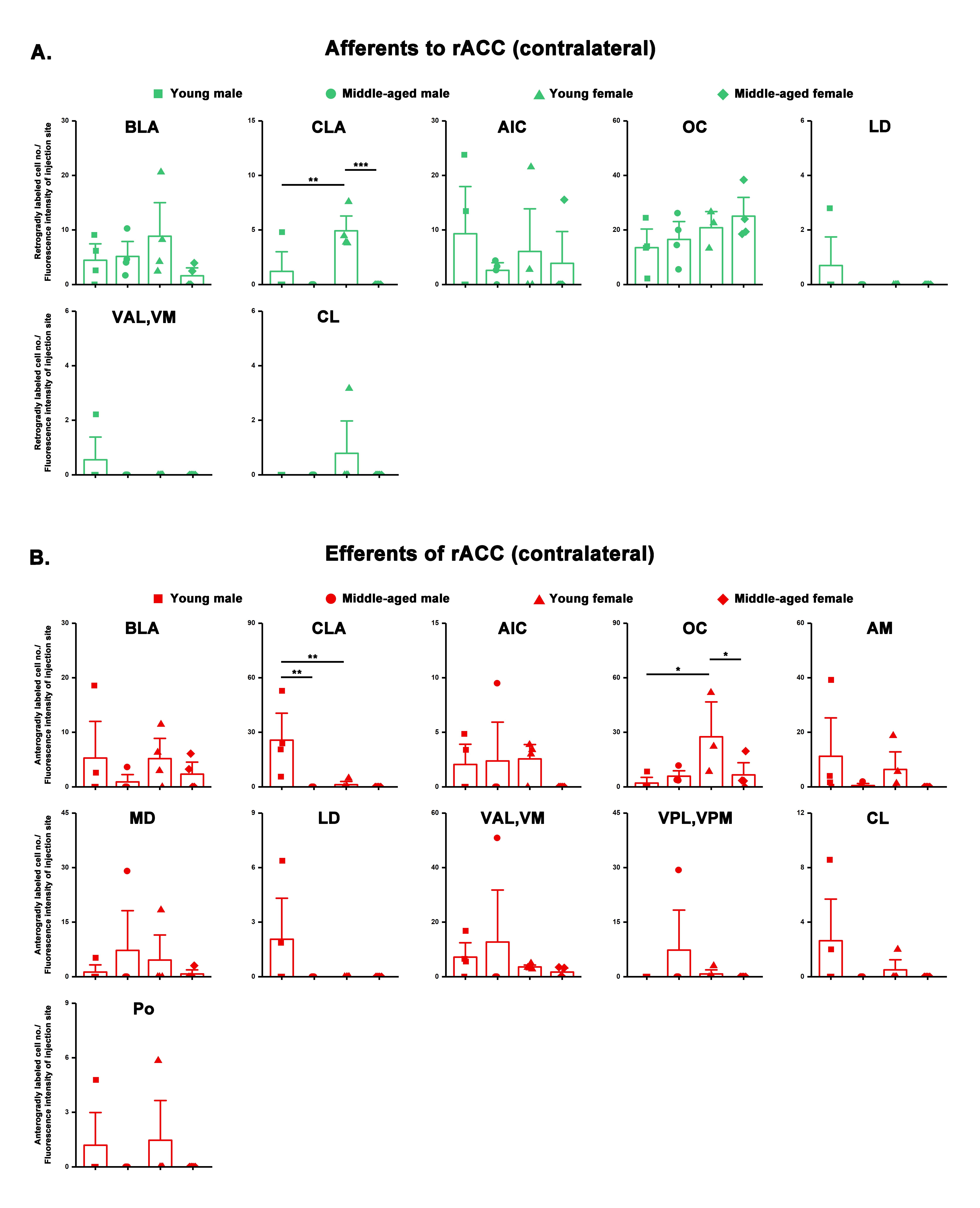

Supplement: Supplementary Figure 2 — Contralateral brain regions with afferent and efferent projections of the rACC in mice of different ages and sexes. n = 4 young males, n = 4 middle-aged males, n = 4 young females, n = 4 young females, and n = 4 middle-aged females. *p < 0.05, **p < 0.01, ***p < 0.001. Error bars represent s.e.m. [file Image_2.jpg]

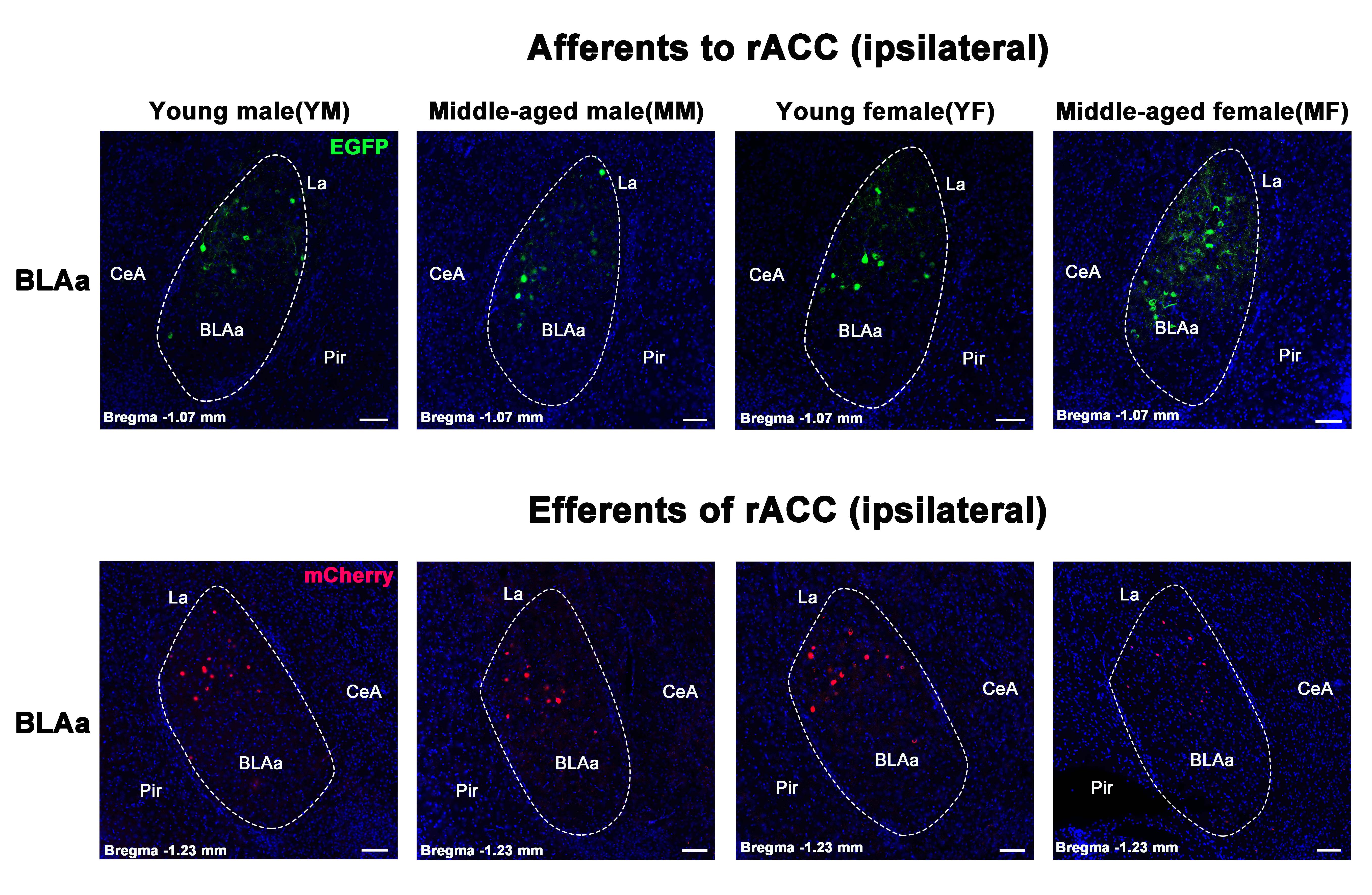

Supplement: Supplementary Figure 3 — Ipsilateral BLAa regions with afferent and efferent projections of the rACC in mice of different ages and sexes. Representative images of retrogradely and anterogradely labeled neurons in the BLAa from the rACC. Scale bar = 100 μm. [file Image_3.jpg]
